# Dickkopf Homolog 3 *(DKK3)* Acts as a Potential Tumor Suppressor in Gallbladder Cancer

**DOI:** 10.3389/fonc.2019.01121

**Published:** 2019-10-29

**Authors:** Kirti Gondkar, Krishna Patel, Geeta V. Patil Okaly, Bipin Nair, Akhilesh Pandey, Harsha Gowda, Prashant Kumar

**Affiliations:** ^1^Institute of Bioinformatics, International Tech Park, Bangalore, India; ^2^Amrita School of Biotechnology, Amrita Vishwa Vidyapeetham, Kollam, India; ^3^Department of Pathology, Kidwai Memorial Institute of Oncology, Bangalore, India; ^4^Manipal Academy of Higher Education, Manipal, India; ^5^Department of Laboratory Medicine and Pathology, Mayo Clinic, Rochester, MN, United States; ^6^Center for Individualized Medicine, Mayo Clinic, Rochester, MN, United States; ^7^Center for Molecular Medicine, National Institute of Mental Health and Neurosciences, Bangalore, India

**Keywords:** gallbladder adenocarcinoma, dickkopf family, biomarker, methylation, tumorigenesis

## Abstract

Gallbladder cancer (GBC) is a common malignancy of biliary tract cancers and its incidence has been rising rapidly worldwide. The prognosis for this disease is dismal as most of the symptoms are non-specific leading to a definitive diagnosis only at a late stage. Loss of *DKK3* gene is associated with a possible tumor suppressor role in human cancers. The role and regulation of DKK3 in GBC have not been studied. We found that DKK3 expression levels were low in GBC patients and cell lines. Treatment of GBC cell lines with demethylating agent 5-Aza- 2'-deoxycytidine enhances its expression, establishing impact of methylation on DKK3 expression. We observed low expression of DKK3 in gallbladder adenocarcinoma tumors and highly invasive GBC cell lines. We showed that overexpression of DKK3 can decrease cell invasion, proliferation, and colony forming ability of GBC cells. Our data thus demonstrated the DKK3 gene is a potential tumor suppressor gene in GBC and aberrant promoter methylation could be involved in its downregulation, which may play a role in the tumorigenesis and aggressiveness of GBC.

## Introduction

Gallbladder cancer (GBC) is the most common, aggressive malignancy of the biliary tract and its incidence has been rising rapidly in recent decades. Both genetic and epigenetic modifications are known to contribute in the tumorigenesis process (1, 2). CpG hypermethylation in the promoter regions of tumor suppressor genes is commonly observed in various cancers. The increasing number of genes inactivated by CpG island hypermethylation is important step in cancer development. Identification and characterization of new gene function would provide a better potential target for diagnosis and treatment of cancers.

Wnt signaling pathway is a hallmark in multiple cancers and its downstream effector regulators are important for cancer progression, including tumor initiation, tumor growth, cell death, and metastasis (3). Aberrant activation of the Wnt pathway either by mutations of intracellular regulators (3) or by altered expression of Wnt proteins or endogenous inhibitors of Wnt signaling has been implicated in a variety of diseases (4). Amongst the protein affected by Wnt signaling, Dickkopf proteins (DKK1,2,4) are known to play a key role (5). DKK1, DKK2, and DKK4 are antagonists of Wnt signaling and interact with Wnt co-receptors, LDL receptor-related protein 5/6 (LRP5/6) and kremen proteins (6, 7).

DKK3 is the only family member, which has the property of unambiguous tumor suppressor and is well-linked with β-catenin pathways and tumor suppression (8, 9). DKK3 expression is ubiquitously detected in mouse and normal human tissues, however it is significantly depleted in various cancer cell types (10). DKK3 silencing because of epigenetic alterations is also reported in multiple cancers (11–13). DKK3 is known to potentiate (14) as well as inhibit (15) Wnt signaling. In a recent study from our group identified a new intracellular gene product originating from the *DKK3* locus and was involved in regulating β-catenin signaling and cellular proliferation (16). Molecularly, GBC involves multiple genetic alterations. Various studies have reported hypermethylation in promoter region of *APC, CDKN2A, CDH1, FHIT, DAPK1, DLC1, MGMT, CDH13, TIMP3, GSTP1*, and *RAR-b2*, which could potentially act as tumor suppressors for GBC (17, 18). However, the role of DKK3 in GBC has not been examined. In the present study, we investigated the role of DKK3 as a tumor suppressor in GBC using *in vitro* model. We used GBC cell lines to perform functional assays to demonstrate the tumor suppressor property of DKK3. Our analysis suggests that DKK3 expression is reduced in GBC tumors as well as cell lines and its overexpression significantly reduce malignant properties in GBC cell lines.

## Materials and Methods

### Cell Culture

Six gallbladder cancer cell lines were used for this study. TGBC2TKB, TGBC24TKB, and G-415 were purchased from RIKEN Bio Resource Center, Ibaraki, Japan. OCUG-1 and NOZ were obtained from Health Science Research Resources Bank, Osaka, Japan. SNU-308 was obtained from Korean Cell Line Bank, Seoul, Korea. TGBC2TKB, SNU-308, TGBC24TKB, G-415, OCUG-1 were cultured in DMEM high glucose, 10% FBS, 1% penicillin/streptomycin. For NOZ DMEM high glucose and DMEM low glucose was used in 1:1 ratio along with 10% FBS, 1% penicillin/streptomycin. Cell lines were maintained in humidified incubator with 5% CO_2_ at 37°C.

### Reverse Transcriptase PCR

Six GBC cell lines upon 70–80% confluency were harvested in Qiazol Lysis Reagent (cat.no. 79306, Qiagen) and RNeasy isolation kit (cat.no. 74104, Qiagen) was used to isolate total RNA from GBC cell lines according to the manufacturer's instructions. cDNA synthesis was carried out with 1 μg of total RNA using Superscript IV First Strand cDNA synthesis Kit (cat.no. 18091050, Invitrogen). PCR was carried out using 1 μL of cDNA, 10 μM of each forward and reverse primers, 50 mM MgCl2, 10 mM dNTP mix, 0.5 Units of Taq polymerase, and PCR buffer in 20 μL reaction volume. Thermal cycling conditions performed to amplify the target sequence are as follows: initial denaturation cycle of 94°C for 2 min, 30 cycles of amplification at with 94°C for 45 s, primer-specific annealing temperature was 57°C and extension temperature at 72°C for 20 s proceeded with final extension of 2 min. RT–PCR was performed using the Rotor-Gene Q (Qiagen). GAPDH gene was used as a loading control. Following DKK3 forward and reverse primers were used for validation 5′-TATGTGTGCAAGCCGACCTT-3′ and 5′-AAAGCACACACCTGGGGAAA-3′Arespectively. A non-template control was run for all reactions. We could not detect any amplification in either of the control reactions. Amplicon sizes were confirmed by running equal volumes of reaction on 2% agarose gel along with 100 bp DNA ladder.

### Western Blotting

The expression of DKK3 across GBC cell lines at protein level was assessed by western blotting. Primary anti DKK3 antibody (cat.no. 126080) was obtained from Abcam. Briefly, protein lysate from 6 GBC cell lines was subjected on 10% SDS-PAGE followed by transferring the proteins on nitrocellulose membrane at cold temperature. Blocking was performed in 5% non-fat dry milk for 40 min followed by overnight primary antibody incubation at 4°C. Next day blots were incubated in anti-rabbit IgG HRP antibody and developed using electrochemiluminescence substrate reagent.

### Transient Transfection of *pCS2-hDKK3-flag*

Three GBC cell lines, OCUG-1, NOZ, and G-415 with relative lower expression of DKK3 were used to determine the optimum concentration of *pCS2-hDKK3-flag* (Addgene cat.no. 15496) along with empty vector *pCS2* as control and X-tremeGENE HP DNA transfection reagent (Roche cat.no. 06366244001). DNA was diluted in opti-MEM to a final concentration of 1 μg plasmid DNA /100 μL medium (0.01 μg/μL) and mixed gently. X-tremeGENE HP DNA transfection reagent was added in 1:2 (DNA:reagent) ratio directly into the medium containing the diluted DNA without coming into contact with the walls of the tube. The complex was mixed gently and incubated for 15 min and added to the cell lines with swirling the wells to ensure even distribution over the entire plate surface. Following transfection, cells were incubated for 72 h and protein expression was measured by western blotting.

### Cell Proliferation Assay

Three GBC Cell lines OCUG-1, NOZ, and G-415 were seeded at a density of 3^*^10^3^ per well in a 96-well plate and were cultured as described above. Cells were then transfected with *pCS2-hDKK3-flag* and then MTT assay was performed for 0, 24, 48, 72, and 96 h. MTT reagent (3-{4,5-dimethylthiazol-2-yl}-2,5-diphenyl-tetrazolium bromide, 5 mg/mL dissolved in DMEM) was added to each well and incubated at 37°C for 4 h. The reaction was stopped by the addition of 70 μL solubilization reagent (50% DMSO + 50% Ethanol) followed by mixing thoroughly to solubilize the crystals. Absorbance values were then measured at 570 and 650 nm as background. All experiments were done in triplicates.

### Cell Invasion Assay

Cell invasion assays were performed in a transwell system using cell culture inserts for 24-well plates with translucent polyethylene terephthalate membrane containing 8 μm pores (BD Biosciences) as described earlier (19). The upper compartment of the culture insert was coated with Matrigel (BD Biosciences). Three GBC cell lines OCUG-1, NOZ, and G-415 after 24 h transfection with *pCS2-hDKK3-flag* were seeded into the transwell chambers in presence of serum-free medium at a density of 2^*^10^4^ along with control set. Complete media was added to the lower compartment and the cells were incubated at 37°C in 5% CO_2_ incubator for 48 h. Post-incubation, the upper surface of the membrane was wiped with a cotton-tip applicator to remove non-migratory cells. Cells that migrated to the lower side of membrane were fixed and stained using 4% methylene blue (Sigma). The number of invaded cells was counted using a light microscope. All experiments were done in triplicates.

### Colony Formation Assay

OCUG-1, NOZ, and G-415 cell lines were seeded in six-well plates at the density of 1.5^*^10^3^ cells/well. Upon small colony formations, cells were transfected with *pCS2-hDKK3-flag* plasmid in 1:2 ratio. Cells were allowed to grow until visible colonies were formed. Re-transfection was done with 1/3rd plasmid concentration at 96 h and colonies were allowed to grow for 7 days. The colonies were fixed with methanol and stained with 4% methylene blue (Sigma). The number of colonies per dish was counted with and without transfection. All experiments were performed in triplicate.

### 5-Aza-2'-deoxcytidine (5-AzaC) Treatment

The OCUG-1, NOZ, G-415 cells were seeded at a density of 1^*^10^5^ cells in a six**-**well culture plates and treated with 5-aza-2'- deoxycytidine (Sigma) at concentrations of 5 and 10 μM daily for 5 and 7 days, respectively. Cell lysates were further subjected to western blotting.

### Immunohistochemical Staining of TMAs

Tissue microarrays (TMAs) were constructed using the paraffin blocks of gallbladder adenocarcinoma (*n* = 71) and cholecystitis (*n* = 19) cases obtained from Cancer Hospital and Research Institute, Gwalior, India with the approval from Institutional Human Ethics Committee and informed consent of the patients. For this, two cores of 2 mm size were taken from each paraffin block and embedded to a recipient paraffin block.

IHC was carried out on both cholecystitis and gallbladder adenocarcinoma cases as reported earlier (20). 1:200 dilution of DKK3 antibody was used across all sections. IHC slides were then observed under the microscope. The immunohistochemical scoring was done by pathologist. The intensity of staining was scored on a grading scale ranging from 0 to 3+, where 0 to 2 represented weak staining, 2+ to 3+ represented moderate, and 3+ represented strong staining.

### Statistical Analysis

To evaluate the difference between control and DKK3 overexpressed groups statistical analysis were performed using Graphpad Prism software (version 6.0) (2-tailed, un-paired *t*-test) and data with *p* < 0.05 were considered statistically significant.

## Results

### Expression of DKK3 Is Downregulated in GBC Tissue and Cell Lines

**Figure 1:**
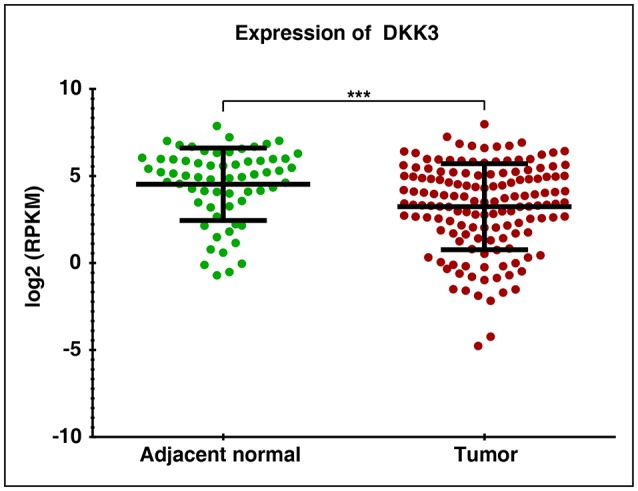
RNA Seq expression profile of DKK3 in gallbladder cancer patients: Downregulation of DKK3 in gallbladder tumors compared to paired normal.

**Figure 2:**
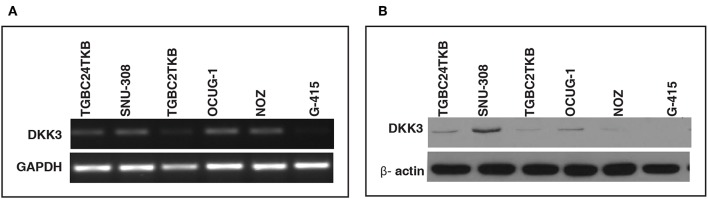
Relative expression of DKK3 in gallbladder cancer cell lines using RT-PCR. **(A)** RT-PCR analysis showed low expression of DKK3 mRNA in GBC cells. **(B)** Western blot analysis depicted relative downregulation of DKK3 from least invasive to highly invasive GBC cell lines.

In order to assess the role of DKK3 in GBC tissues, we used our unpublished RNA seq data (21). The sequencing was done on tumors and paired normal. Expression of DKK3 gene was quantified in FPKM unit and boxplot was generated using non-parametric unpaired two-tailed *t*-test using Graphpad Prism. Our results suggest that expression of DKK3 is significantly reduced in tumor samples compared to adjacent normal patient samples (Figure 1). To further validate the expression levels of DKK3, we performed RT-PCR and western blot across 6 GBC cell lines. These six cell lines are well**-**characterized based on their invasiveness ability from non-invasive to highly invasive by Subbannayya et al. (22). TGBC24TKB is non-invasive, SNU-308 and TGBC2TKB are less invasive, OCUG-1 and NOZ been moderately invasive and G-415 characterized as highly invasive cell lines. We observed low to negligible level of DKK3 expression in all the cell lines at the transcript level in all the cell lines (Figure 2A). Further, we checked the expression of DKK3 at protein level. Our western blot analysis suggests prominent expression of DKK3 in TGBC24TKB, SNU-308 and OCUG-1 with highest in SNU-308 cell line (Figure 2B). We also observe a slight expression of DKK3 in TGBC2TKB and NOZ. However, G-415 cell line showed a negligible expression of DKK3. Our results indicate that the expression of DKK3 is significantly down regulated in highly invasive GBC cell line G-415 in comparison to non-invasive or less invasive cell lines.

### DKK3 Overexpression Reduces Proliferation of Gallbladder Cancer Cells

**Figure 3:**
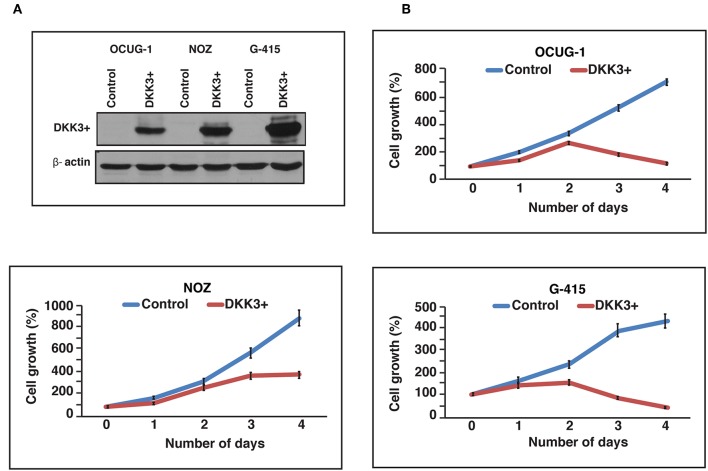
DKK3 overexpression affects proliferation in GBC cells. **(A)** Transient transfection of DKK3 in OCUG-1, NOZ and G-415 shows the overexpression. **(B)** Proliferation of GBC cells is reduced upon DKK3 overexpression.

We further studied the effects of DKK3 overexpression with regard to cellular proliferation in GBC cell lines. To characterize the tumor suppressor function of DKK3, we chose 3 cell lines, which showed reduced DKK3 expression and were invasive in nature. Two moderately invasive and one highly invasive GBC cell lines OCUG-1, NOZ, and G-415 were optimized for transient transfection of DKK3 (Figure 3A). Upon overexpression of DKK3 in these cell lines, we analyzed the change in metabolic activity of these cells. The reduction of yellow tetrazolium salt MTT, to purple colored compound only occurs in metabolically active cells. Based on spectrophotometric analysis, we observed a decrease in metabolic ability in all the three cell lines (Figure 3B) compared to control cells. Upon DKK3 overexpression, we were able to see the decrease in cell growth from day 2 onwards for OCUG-1 and G-415 cells and day 3 for NOZ cells. Our results showed that DKK3 overexpression decreases cell proliferation in all the 3 GBC cell lines.

### DKK3 Overexpression Reduces Invasiveness of Gallbladder Cancer Cells

**Figure 4:**
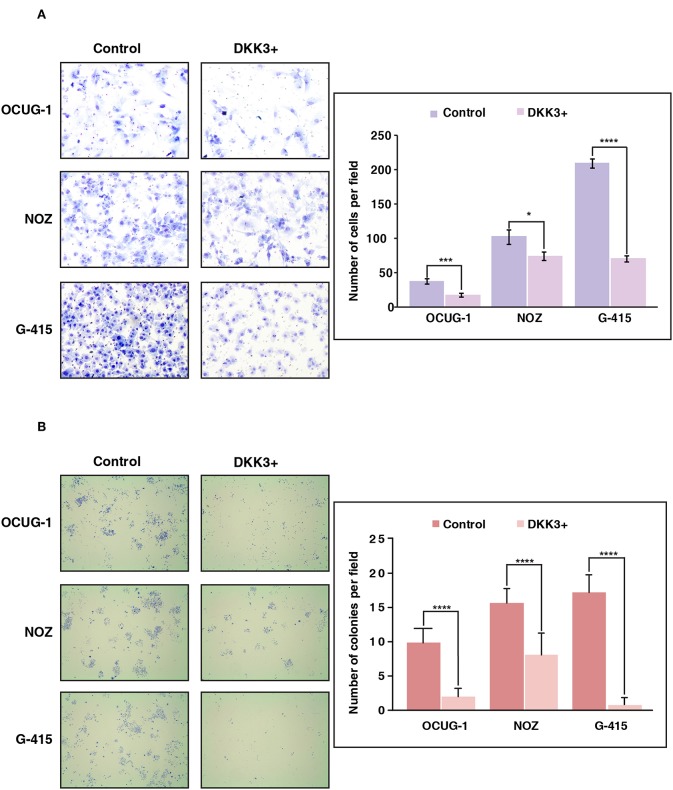
Overexpression of DKK3 reduces cell invasion and colony forming ability *in vitro*. **(A)** DKK3 overexpression shows reduced invasiveness as assessed by Matrigel chamber assay. Invaded cells were counted after methylene blue staining. Graph depicting number of invaded cells per field with and without DKK3 overexpression **(B)** DKK3 overexpression decreases the colony forming ability of GBC cells. Colonies were fixed and stained with methylene blue. Graph depicting number of colonies per field with and without DKK3 overexpression.

We further sought to study the potential role of DKK3 overexpression in regulating invasive ability across moderately invasive and highly invasive cell lines. Proliferating cells attempt to evade from the primary tumor site, to reach to distant organ the process involves local invasion of cells by degrading extracellular matrix proteins and crossing the basement membrane. We used matrigel assisted boyden chamber invasion assay to understand the effect of DKK3 on invasive ability of cells. Matrigel acts as an attachment, which resembles to extracellular matrix environment. Upon overexpression of DKK3, we observed a significant decrease in the invasive ability of highly invasive G-415 cell line (*p* < 0.0001) and moderately invasive OCUG-1 (*p* = 0.0007) while NOZ showed decreased invasiveness with less significance (*p* = 0.0371) (Figure 4A). These results suggest that DKK3 significantly reduces the invasiveness in GBC cell lines.

### Colony Forming Ability of GBC Cells Were Decreased Upon DKK3 Overexpression

To understand if DKK3 has an effect on the ability of a single cancerous cell to proliferate and differentiate, we checked the colony forming ability of cells upon DKK3 overexpression. The potential of cells to proliferate and differentiate in isolated environment can be assessed by clonogenic assay or colony formation assay. Upon DKK3 overexpression in 3 GBC cell lines, OCUG-1, NOZ, G-415, we observed the ability of these cell lines to form colonies was decreased significantly (*p* < 0.0001) (Figure 4B), suggesting DKK3 inhibits the capacity of single cells to proliferate and could act as potential tumor suppressor in GBC.

### DKK3 Expression Is Variable Across Cholecystitis and Gallbladder Adenocarcinoma Tissues

**Figure 5:**
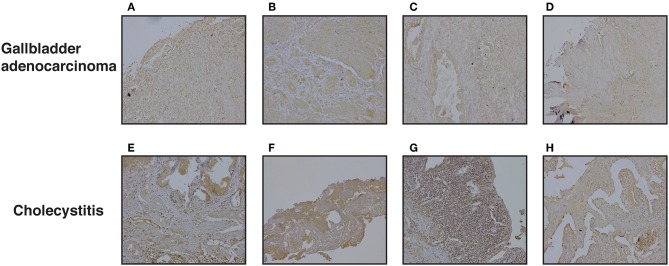
Representative IHC images depicting low expression of DKK3 in gallbladder cancer compared to high expression in cholecystitis sections **(A)** represent GBC grade 3, weak staining **(B)** represent GBC grade 3, weak staining **(C)** represent GBC grade 2, weak staining **(D)** represent GBC grade 2, weak staining **(E)** represent cholecystitis strong staining **(F)** represent cholecystitis moderate staining **(G)** represent cholecystitis moderate staining **(H)** represent cholecystitis moderate staining.

We further evaluated DKK3 expression by immunohistochemistry. Tissue microarray-based validation was carried out on 71 GBC and 19 cholecystitis tissue sections. DKK3 was found to be predominantly localized in cytoplasm. The tumor tissues belong from grade 1 (*n* = 7), grade 2 (*n* = 34), and grade 3 (*n* = 30) gallbladder adenocarcinoma cases. We observed a variable staining pattern of DKK3 across GBC. In cholecystitis, 68.4% cases showed weak staining intensity while 31% showed strong intensity. Grade wise, we observed 100, 76.5, and 63.3% showed negative to weak staining in grade 1, grade 2, and grade 3 cases, respectively while 14.7 and 20% showed strong staining in grade 2 and grade 3 cases, respectively. The results of immunohistochemical staining are provided in Table 1A. Representative staining patterns for each staining intensity of GBC and cholecystitis are shown in Figure 5.

**Table 1:** Immunohistochemical scores of DKK3 in gallbladder tissue.

**Staining intensity**	**Grade 1** **(%)**	**Grade 2** **(%)**	**Grade 3** **(%)**	**Cholecystitis** **(%)**
**A**				
Weak	100	76.5	63.3	68.4
Moderate	0	8.8	16.7	0.0
Strong	0	14.7	20.0	31.6
**Staining intensity**		**Tumor (%)**		**Cholecystitis (%)**
**B**				
Weak		74.6		68.4
Moderate		11.3		0.0
Strong		14.1		31.6

*(A) Grade wise distribution of DKK3 expression in GBC (B) Summary of DKK3 expression in GBC*.

### 5-Aza- 2'-deoxycytidine Restores DKK3 Expression

**Figure 6:**
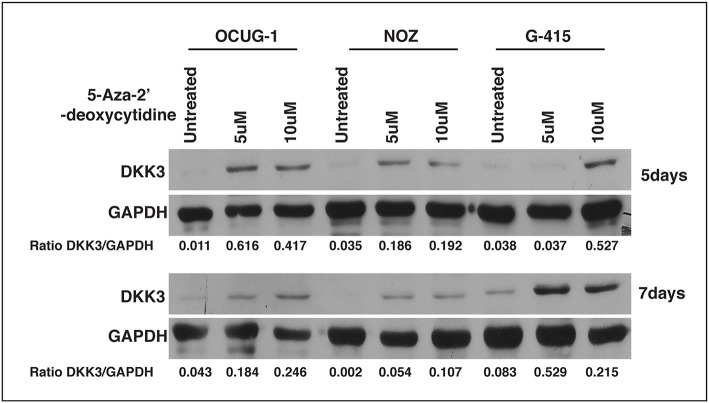
Effect of 5-Aza- 2'-deoxycytidine, an DNMT inhibitor on DKK3 expression. DKK3 expression is restored upon treatment with 5AzaC in OCUG-1, NOZ, and G-415 cells at 5 and 7 days.

We observed that DKK3 expression was negligible in invasive gallbladder cancer cell lines OCUG-1, NOZ, and G415. We further assessed the role of promoter hypermethylation of DKK3 gene. We treated GBC cell lines with potent demethylating agent 5-Aza- 2'-deoxycytidine at 5 and 10 μM concentrations for 5 days and 7 days, respectively. Our results indicate that upon treating the cells with 5-Aza- 2'-deoxycytidine, the expression of DKK3 is restored. This suggests promoter hypermethylation may play a key role in silencing of DKK3 expression in GBC cell lines (Figure 6).

## Discussion

GBC is a rare neoplasia with limited understanding of cellular changes associated with disease. Inflammation of gallbladder also known as cholecystitis, and formation of gallstones are major risk factors associated with formation of gallbladder malignancies (23). Various high throughput studies have reported complex interplay of genomic, epigenomics, transcriptomics, and proteomic profile in gallbladder carcinoma. However, few studies have investigated molecular mechanisms associated with gallbladder carcinogenesis. DKK3 belongs to DKK family comprising of DKK1, DKK2, and DKK4, which are known as a potent antagonist of Wnt/β-catenin signaling pathway (24). Unlike other members of DKK family, role of DKK3 in Wnt signaling is still elusive however it is reported to have both oncogenic and tumor suppressive property (25–34) (Supplementary Table 1). DKK3 is reported as overexpressed in esophageal adenocarcinoma (35), head and neck squamous cell carcinoma (36), and hepatoblastoma (37); whereas studies have reported its downregulated in pancreatic cancer (38), clear cell renal cell carcinoma (39), gastric cancer (40), basal subtype of breast cancer (41), and melanoma (42). We also observed statistically significant reduced expression of DKK3 in unpublished RNA-Seq datasets of GBC cell lines and patients. To validate potential tumor suppressor property of DKK3, we further assessed expression of DKK3 on mRNA and protein level in 6 GBC cell lines using RT-PCR and western blot, respectively. Despite of miniscule mRNA expression of DKK3 in 5 cell lines except highly invasive G-415 cell line; protein expression was notably observed in TGBC24TKB, SNU-308, and OCUG-1, reduced expression in TGBC2TKB and NOZ and negligible expression in G-415. Here we demonstrate possible association of reduced expression of DKK3 with high-grade gallbladder carcinoma.

We further investigated functional implications of DKK3 downregulation using two moderately invasive characterized GBC cell lines NOZ and OCUG-1 and one highly invasive GBC cell line G-415. We demonstrate that transient overexpression of DKK3 evidently minimized cell proliferation and colony formation rate in all the three cell lines. Upon transient DKK3 overexpression, we observed moderate decrease in invasiveness of NOZ cells, however significant decrease in invasiveness was observed in OCUG-1 and G-415 cells. Equivalent tumor suppressive property on DKK3 downregulation is exhibited in hepatocellular carcinoma, cervical cancer (43), gastric cancer (44), and non-small-cell lung carcinomas cells (45). In coherence with retrospective observation from different cancers, we further confirm functionally repressed DKK3 in gallbladder carcinoma cells.

Gene promoter hypermethylation of DKK3 is reported in malignant biliary stricture (11), gastrointestinal cancer (46), prostate cancer (47), and acute lymphoblastic leukemia (48) leading to a speculation that epigenetic alteration of DKK3 gene promoter region may be major regulatory mechanism commonly associated with tumorigenesis. However, the exact mechanism for downregulation of DKK3 in GBC remains to be studied. Methylation of DKK3 gene family have been correlated with carcinogenesis of several human malignancies (9, 26, 49). Dkk3 is also reported to be expressed in normal human tissues (50, 51). However, the role of DKK3 gene and its epigenetic regulation has not been studied in GBC.

In accordance with our study, we examined the relationship between the expression and methylation of DKK3. DKK3 expression levels were restored after treating the cells with 5-Aza- 2'-deoxycytidine in all 3 GBC cell lines, suggesting that promoter hypermethylation could be one of the regulatory mechanisms for DKK3 inactivation in GBC cells. We compared 5-Aza- 2'-deoxycytidine alone to two different concentrations. Thus, hypermethylation may be involved in silencing DKK3 expression in GBC. In the future, the mechanisms of DKK3 regulation requires further study on tissue samples as well. We further correlate the expression of DKK3 in GBC by IHC validation on total 90 patient samples. We observed about 74.6% of gallbladder adenocarcinoma cases showed weak staining intensity, 11.3% showed moderate intensity, and 14.1% showed strong intensity (Table 1B). Gallbladder adenocarcinoma and cholecystitis cases exhibited weak IHC staining suggesting intertumor heterogeneity. We do not observe decrease in expression of DKK3 with increase in grade of tumors.

In summary, we identified DKK3 as a novel functional tumor suppressor gene in GBC. DKK3 contributes to the suppression of tumorigenesis by decreasing cell proliferation, invasion and colony formation. Expression and methylation levels can be further harnessed as potential biomarkers diagnostic marker and may have a prognostic importance in the risk stratification in the patients of GBC. However, validation in larger patient cohort is warranted.

## Data Availability Statement

All datasets generated for this study are included in the manuscript/Supplementary Files.

## Ethics Statement

The studies involving human participants were reviewed and approved by Cancer Hospital and Research Institute, Gwalior. The patients/participants provided their written informed consent to participate in this study.

## Author Contributions

PK initiated, conceptualized, investigated, supervised, and acquired funding for the project. KG, KP, and GP performed experiments and analyzed data. KG, KP, and PK wrote the manuscript. HG, AP, and PK helped in acquiring resources, reviewing, and editing manuscript. BN, HG, AP, and PK supervised the project. All authors have read and approved the final version of the manuscript.

### Conflict of Interest

The authors declare that the research was conducted in the absence of any commercial or financial relationships that could be construed as a potential conflict of interest.

## References

[1] Tewari M Agarwal A Mishra RR Meena RN Shukla HS. Epigenetic changes in carcinogenesis of gallbladder. Indian J Surg Oncol. (2013) 4:356–61. 10.1007/s13193-013-0240-0 24426757 PMC3890022

[2] Barreto SG Dutt A Chaudhary A. A genetic model for gallbladder carcinogenesis and its dissemination. Ann Oncol. (2014) 25:1086–97. 10.1093/annonc/mdu006 24705974 PMC4037856

[3] MacDonald BT Tamai K He X. Wnt/beta-catenin signaling: components, mechanisms, and diseases. Dev Cell. (2009) 17:9–26. 10.1016/j.devcel.2009.06.016 19619488 PMC2861485

[4] Pinzone JJ Hall BM Thudi NK Vonau M Qiang YW Rosol TJ. The role of Dickkopf-1 in bone development, homeostasis, and disease. Blood. (2009) 113:517–25. 10.1182/blood-2008-03-145169 18687985 PMC2628360

[5] Niehrs C. Function and biological roles of the Dickkopf family of Wnt modulators. Oncogene. (2006) 25:7469–81. 10.1038/sj.onc.1210054 17143291

[6] Davidson G Mao B del Barco Barrantes I Niehrs C. Kremen proteins interact with Dickkopf1 to regulate anteroposterior CNS patterning. Development. (2002) 129:5587–96. 10.1242/dev.00154. 12421700

[7] Mao B Wu W Davidson G Marhold J Li M Mechler BM. Kremen proteins are Dickkopf receptors that regulate Wnt/beta-catenin signalling. Nature. (2002) 417:664–7. 10.1038/nature756 12050670

[8] Hoang BH Kubo T Healey JH Yang R Nathan SS Kolb EA. Dickkopf 3 inhibits invasion and motility of Saos-2 osteosarcoma cells by modulating the Wnt-beta-catenin pathway. Cancer Res. (2004) 64:2734–9. 10.1158/0008-5472.CAN-03-1952 15087387

[9] Veeck J Dahl E. Targeting the Wnt pathway in cancer: the emerging role of Dickkopf-3. Biochim Biophys Acta. (2012) 1825:18–28. 10.1016/j.bbcan.2011.09.003 21982838

[10] Zhang K Watanabe M Kashiwakura Y Li SA Edamura K Huang P. Expression pattern of REIC/Dkk-3 in various cell types and the implications of the soluble form in prostatic acinar development. Int J Oncol. (2010) 37:1495–501. 10.3892/ijo_00000802 21042718

[11] Zhang Y Yang B Du Z Gao YT Wang YJ Jing X. Identification and validation of specific methylation profile in bile for differential diagnosis of malignant biliary stricture. Clin Biochem. (2010) 43:1340–4. 10.1016/j.clinbiochem.2010.08.013 20727349

[12] Xiang T Li L Yin X Zhong L Peng W Qiu Z. Epigenetic silencing of the WNT antagonist Dickkopf 3 disrupts normal Wnt/beta-catenin signalling and apoptosis regulation in breast cancer cells. J Cell Mol Med. (2013) 17:1236–46. 10.1111/jcmm.12099 23890219 PMC4159020

[13] Liang L He H Lv R Zhang M Huang H An Z. Preliminary mechanism on the methylation modification of Dkk-1 and Dkk-3 in hepatocellular carcinoma. Tumour Biol. (2015) 36:1245–50. 10.1007/s13277-014-2750-y 25344678

[14] Nakamura RE Hackam AS. Analysis of Dickkopf3 interactions with Wnt signaling receptors. Growth Factors. (2010) 28:232–42. 10.3109/08977191003738832 20370576 PMC2950226

[15] Caricasole A Ferraro T Iacovelli L Barletta E Caruso A Melchiorri D. Functional characterization of WNT7A signaling in PC12 cells: interaction with A FZD5 x LRP6 receptor complex and modulation by Dickkopf proteins. J Biol Chem. (2003) 278:37024–31. 10.1074/jbc.M300191200 12857724

[16] Leonard JL Leonard DM Wolfe SA Liu J Rivera J Yang M The Dkk3 gene encodes a vital intracellular regulator of cell proliferation. PLoS ONE. (2017) 12:e0181724 10.1371/journal.pone.0181724 28738084 PMC5524345

[17] Roa JC Anabalon L Roa I Melo A Araya JC Tapia O. Promoter methylation profile in gallbladder cancer. J Gastroenterol. (2006) 41:269–75. 10.1007/s00535-005-1752-3 16699861

[18] Garcia P Manterola C Araya JC Villaseca M Guzman P Sanhueza A. Promoter methylation profile in preneoplastic and neoplastic gallbladder lesions. Mol Carcinog. (2009) 48:79–89. 10.1002/mc.20457 18543280

[19] Gondkar K Patel K Krishnappa S Patil A Nair B Sundaram GM. E74 like ETS transcription factor 3 (ELF3) is a negative regulator of epithelial- mesenchymal transition in bladder carcinoma. Cancer Biomark. (2019) 25:223–32. 10.3233/CBM-190013 31104013 PMC13082411

[20] Deb B Puttamallesh VN Gondkar K Thiery JP Gowda H Kumar P. Phosphoproteomic profiling identifies aberrant activation of integrin signaling in aggressive non-type bladder carcinoma. J Clin Med. (2019) 8:E703. 10.3390/jcm8050703 31108958 PMC6572125

[21] Pandey A Stawiski EW Durinck S Gowda H Goldstein LD Barbhuiya MA ELF3 is a significantly mutated gallbladder cancer vaccine candidate. Manuscript under review.

[22] Subbannayya T Leal-Rojas P Barbhuiya MA Raja R Renuse S Sathe G. Macrophage migration inhibitory factor - a therapeutic target in gallbladder cancer. BMC Cancer. (2015) 15:843. 10.1186/s12885-015-1855-z 26530123 PMC4632274

[23] Goetze TO. Gallbladder carcinoma: prognostic factors and therapeutic options. World J Gastroenterol. (2015) 21:12211–7. 10.3748/wjg.v21.i43.12211 26604631 PMC4649107

[24] Semenov MV Tamai K Brott BK Kuhl M Sokol S He X. Head inducer Dickkopf-1 is a ligand for Wnt coreceptor LRP6. Curr Biol. (2001) 11:951–61. 10.1016/s0960-9822(01)00290-1 11448771

[25] Katase N. Gunduz M. Beder L. Gunduz E. Lefeuvre M. Hatipoglu O. F.. (2008). Deletion at Dickkopf (dkk)-3 locus (11p15.2) is related with lower lymph node metastasis and better prognosis in head and neck squamous cell carcinomas. Oncol Res. 17, 273–282. 10.3727/096504008786991594 19192722

[26] Veeck J Bektas N Hartmann A Kristiansen G Heindrichs U Knuchel R. Wnt signalling in human breast cancer: expression of the putative Wnt inhibitor Dickkopf-3 (DKK3) is frequently suppressed by promoter hypermethylation in mammary tumours. Breast Cancer Res. (2008) 10:R82. 10.1186/bcr2151 18826564 PMC2614517

[27] Yue W Sun Q Dacic S Landreneau RJ Siegfried JM Yu J. Downregulation of Dkk3 activates beta-catenin/TCF-4 signaling in lung cancer. Carcinogenesis. (2008) 29:84–92. 10.1093/carcin/bgm267 18048388

[28] Lee EJ Jo M Rho SB Park K Yoo YN Park J. Dkk3, downregulated in cervical cancer, functions as a negative regulator of beta-catenin. Int J Cancer. (2009) 124:287–97. 10.1002/ijc.23913 19003969

[29] Zhang Y Dong WG Yang ZR Lei XF Luo HS. [Expression of Dickkopf-3 in esophageal squamous cell carcinoma]. Zhonghua Nei Ke Za Zhi. (2010) 49:325–7. 20627041

[30] Liu JB Qiang FL Dong J Cai J Zhou SH Shi MX. Plasma DNA methylation of Wnt antagonists predicts recurrence of esophageal squamous cell carcinoma. World J Gastroenterol. (2011) 17:4917–21. 10.3748/wjg.v17.i44.4917 22171134 PMC3235636

[31] Zenzmaier C Heitz M Klocker H Buck M Gardiner RA Berger P. Elevated levels of Dickkopf-related protein 3 in seminal plasma of prostate cancer patients. J Transl Med. (2011) 9:193. 10.1186/1479-5876-9-193 22071168 PMC3240830

[32] Zenzmaier C Hermann M Hengster P Berger P. Dickkopf-3 maintains the PANC-1 human pancreatic tumor cells in a dedifferentiated state. Int J Oncol. (2012) 40:40–6. 10.3892/ijo.2011.1180 21879258

[33] Kloten V Becker B Winner K Schrauder MG Fasching PA Anzeneder T. Promoter hypermethylation of the tumor-suppressor genes ITIH5, DKK3, and RASSF1A as novel biomarkers for blood-based breast cancer screening. Breast Cancer Res. (2013) 15:R4. 10.1186/bcr3375 23320751 PMC3672828

[34] Yin DT Wu W Li M Wang QE Li H Wang Y. DKK3 is a potential tumor suppressor gene in papillary thyroid carcinoma. Endocr Relat Cancer. (2013) 20:507–14. 10.1530/ERC-13-0053 23702469

[35] Wang Z Lin L Thomas DG Nadal E Chang AC Beer DG. The role of Dickkopf-3 overexpression in esophageal adenocarcinoma. J Thorac Cardiovasc Surg. (2015) 150:377–85 e372. 10.1016/j.jtcvs.2015.05.006 26093488 PMC4515149

[36] Katase N Nishimatsu SI Yamauchi A Yamamura M Terada K Itadani M. DKK3 Overexpression increases the malignant properties of head and neck squamous cell carcinoma cells. Oncol Res. (2018) 26:45–58. 10.3727/096504017X14926874596386 28470144 PMC7844562

[37] Pei Y Yao Q Yuan S Xie B Liu Y Ye C. GATA4 promotes hepatoblastoma cell proliferation by altering expression of miR125b and DKK3. Oncotarget. (2016) 7:77890–901. 10.18632/oncotarget.12839 27788486 PMC5363629

[38] Uchida D Shiraha H Kato H Nagahara T Iwamuro M Kataoka J. Potential of adenovirus-mediated REIC/Dkk-3 gene therapy for use in the treatment of pancreatic cancer. J Gastroenterol Hepatol. (2014) 29:973–83. 10.1111/jgh.12501 24372695

[39] Guo CC Zhang XL Yang B Geng J Peng B Zheng JH. Decreased expression of Dkk1 and Dkk3 in human clear cell renal cell carcinoma. Mol Med Rep. (2014) 9:2367–73. 10.3892/mmr.2014.2077 24676838

[40] Park JM Kim MK Chi KC Kim JH Lee SH Lee EJ. Aberrant loss of dickkopf-3 in gastric cancer: can it predict lymph node metastasis preoperatively? World J Surg. (2015) 39:1018–25. 10.1007/s00268-014-2886-3 25604390

[41] Lorsy E Topuz AS Geisler C Stahl S Garczyk S von Stillfried S. Loss of Dickkopf 3 promotes the tumorigenesis of basal breast cancer. PLoS ONE. (2016) 11:e0160077. 10.1371/journal.pone.0160077 27467270 PMC4965070

[42] Huo J Zhang Y Li R Wang Y Wu J Zhang D. Upregulated microRNA-25 mediates the migration of melanoma cells by targeting DKK3 through the WNT/beta-catenin pathway. Int J Mol Sci. (2016) 17:E1124. 10.3390/ijms17111124 27801786 PMC5133768

[43] Hsieh SY Hsieh PS Chiu CT Chen WY. Dickkopf-3/REIC functions as a suppressor gene of tumor growth. Oncogene. (2004) 23:9183–9. 10.1038/sj.onc.1208138 15516983

[44] Sato H Suzuki H Toyota M Nojima M Maruyama R Sasaki S. Frequent epigenetic inactivation of DICKKOPF family genes in human gastrointestinal tumors. Carcinogenesis. (2007) 28:2459–66. 10.1093/carcin/bgm178 17675336

[45] Tsuji T Nozaki I Miyazaki M Sakaguchi M Pu H Hamazaki Y. Antiproliferative activity of REIC/Dkk-3 and its significant down-regulation in non-small-cell lung carcinomas. Biochem Biophys Res Commun. (2001) 289:257–63. 10.1006/bbrc.2001.5972 11708809

[46] Maehata T Taniguchi H Yamamoto H Nosho K Adachi Y Miyamoto N. Transcriptional silencing of Dickkopf gene family by CpG island hypermethylation in human gastrointestinal cancer. World J Gastroenterol. (2008) 14:2702–14. 10.3748/wjg.14.2702 18461655 PMC2709050

[47] Lodygin D Epanchintsev A Menssen A Diebold J Hermeking H. Functional epigenomics identifies genes frequently silenced in prostate cancer. Cancer Res. (2005) 65:4218–27. 10.1158/0008-5472.CAN-04-4407 15899813

[48] Roman-Gomez J Jimenez-Velasco A Agirre X Castillejo JA Navarro G Barrios M. Transcriptional silencing of the Dickkopfs-3 (Dkk-3) gene by CpG hypermethylation in acute lymphoblastic leukaemia. Br J Cancer. (2004) 91:707–13. 10.1038/sj.bjc.6602008 15226763 PMC2364778

[49] van der Meide WF Snellenberg S Meijer CJ Baalbergen A Helmerhorst TJ van der Sluis WB Promoter methylation analysis of WNT/beta-catenin signaling pathway regulators to detect adenocarcinoma or its precursor lesion of the cervix. Gynecol Oncol. (2011) 123:116–22. 10.1016/j.ygyno.2011.06.015 21726894

[50] Hermann M Pirkebner D Draxl A Berger P Untergasser G Margreiter R. Dickkopf-3 is expressed in a subset of adult human pancreatic beta cells. Histochem Cell Biol. (2007) 127:513–21. 10.1007/s00418-007-0278-6 17347849

[51] Du G Kataoka K Sakaguchi M Abarzua F Than SS Sonegawa H. Expression of REIC/Dkk-3 in normal and hyperproliferative epidermis. Exp Dermatol. (2011) 20:273–7. 10.1111/j.1600-0625.2010.01244.x 21323747

